# The StABI5-*StCAT1* Module Regulates Potato Resistance to Early Blight Through the ROS Signaling Pathway

**DOI:** 10.3390/biology15151250

**Published:** 2026-07-29

**Authors:** Bingbing Li, Mengxiang Shi, Yunjing Zhang, Xin Liu, Zhijiang Zhang, Yan Feng, Zhihui Yang, Qian Li

**Affiliations:** 1 College of Plant Protection, Hebei Agricultural University, Baoding 071001, China; 2 Chengde Academy of Agricultural and Forestry Sciences, Chengde 067000, China; 3 Center for Dryland Agriculture Research, Hebei North University, Zhangjiakou 075000, China

**Keywords:** *StCAT1*, *Alternaria solani*, reactive oxygen species, StABI5

## Abstract

Potato early blight is a fungal disease caused by *Alternaria solani* that seriously affects potato production. Catalase (CAT), an important antioxidant enzyme in plants, regulates the basal immune response to necrotrophic pathogens by decomposing hydrogen peroxide (H_2_O_2_); however, its role in potato early blight remains unclear. The results revealed that using a H_2_O_2_ biosensor for transient overexpression in *Nicotiana benthamiana*, we found that *A. solani* infection significantly induced sustained H_2_O_2_ accumulation in tobacco leaves. Exogenous application of H_2_O_2_ to potato leaves followed by inoculation with *A. solani* showed that elevated H_2_O_2_ levels promoted infection by the pathogen. *StCAT1* expression was upregulated upon *A. solani* infection. Silencing *StCAT1* in potato reduced antioxidant enzyme activities (SOD, PAL, CAT) and disease resistance, accompanied by elevated H_2_O_2_ accumulation. Overexpression of *StCAT1* enhanced antioxidant enzyme activities and disease resistance while decreasing H_2_O_2_ levels. Additionally, StABI5 was identified as an upstream transcription factor that activates *StCAT1* expression, and silencing *StABI5* compromised potato resistance to early blight. In conclusion, this study confirms that StCAT1 acts as a positive regulatory factor and mediates potato resistance to early blight through the StABI5-*StCAT1* signaling pathway, providing scientific basis and genetic resources for disease-resistant breeding.

## Simple Summary

Potato early blight caused by *Alternaria solani* poses a serious threat to potato yield and quality. Catalase regulates plant immunity against necrotrophic pathogens, yet its function in early blight remains unclear. In this study, *A. solani* infection induces hydrogen peroxide accumulation in plants, and exogenous H_2_O_2_ application markedly accelerates early blight infection. Furthermore, we identified *StCAT1* as a positive regulator of resistance to early blight in potato, with its expression significantly upregulated following *A. solani* induction. Silencing *StCAT1* impaired antioxidant capacity and disease resistance, while overexpression exerted the opposite effect. Additionally, StABI5 was identified as an upstream transcription factor that activates *StCAT1* expression, and silencing *StABI5* compromised potato resistance to early blight. These results indicate that StABI5 can activate the expression of *StCAT1* and by mediating the reactive oxygen species (ROS) signaling pathway regulates potato resistance to early blight, which provides a theoretical basis and genetic resources for the molecular breeding of potato resistance to early blight.

## 1. Introduction

Potato early blight, the second most prevalent disease in China after late blight, is caused by *Alternaria solani*. *A. solani* is a typical necrotrophic fungal pathogen [1]. This disease is widely distributed and has been reported in potato-growing regions worldwide, with an average annual affected area of approximately 900,000 hectares, causing severe yield losses in major potato-producing areas [2]. Currently, disease management primarily relies on chemical fungicides [3], which rapidly suppress disease spread. However, these chemicals can severely impact soil ecosystems, cause environmental pollution, and lead to pathogen resistance through prolonged use. This disrupts soil microbial communities, driving up control costs year after year [4]. Potato varieties resistant to early blight, developed through genetic engineering, can effectively control the disease without the use of chemical fungicides. Therefore, identifying novel potato genes that confer resistance to early blight and elucidating their functional mechanisms are of critical importance.

Catalase (CAT), as a key regulator of plant disease resistance, influences plant resistance to pathogens through multi-level interactions with plant defense responses [5,6]. During plant–microbe interactions, inducers released by pathogens can be recognized by the plant immune system, triggering bursts of reactive oxygen species such as H_2_O_2_ [7]. Furthermore, as a core antioxidant enzyme, CAT, catalyzes the decomposition of H_2_O_2_ into water and oxygen, thereby regulating redox homeostasis and mitigating oxidative damage while maintaining effective defense responses [8,9]. Additionally, *CAT* modulates defense gene expression by interacting with MAPK/NLR signaling pathways, influencing plant resistance strategies against pathogens [10,11]. Simultaneously, this enzyme interacts with defense hormone pathways such as jasmonic acid (JA) and salicylic acid (SA) [12,13], influencing plant-specific defense responses to pathogens with distinct nutritional modes by modulating JA/SA signaling balance. Finally, changes in catalase activity can significantly affect the intensity of pathogen infection [14,15]. Previous studies have shown that a highly sensitive fluorescent probe based on a rhodol structure has been successfully applied for the detection of hydrogen peroxide in living cells and zebrafish [16]. A novel near-infrared fluorescent probe, LDMD-B, enables selective detection of H_2_O_2_ through a boronate cleavage mechanism and has been applied in an Alzheimer’s disease model [17]. A H_2_O_2_-activated CRISPR/Cas12a imaging strategy (A-BO-CRISPR) enables real-time in vivo fluorescence imaging of oxidative stress markers, with a limit of detection as low as 0.64 μM and a response time of only a few minutes [18]. However, there are still relatively few studies on hydrogen peroxide probes in plants.

Reactive oxygen species primarily include H_2_O_2_, O2∙^−^ and ∙OH. As crucial components of plant defense against biotic stress, ROS function as signaling molecules to activate defense responses or directly eliminate pathogens via their potent oxidizing properties [19]. However, excessive accumulation of ROS can cause oxidative damage to plant cells. Consequently, the dynamic balance of ROS concentrations dictates the efficacy of disease resistance, requiring the antioxidant system to maintain homeostatic thresholds [20,21]. Moreover, ROS may exert contrasting effects on plant resistance to pathogens with different trophic lifestyles [22]. Necrotrophic pathogens actively utilize ROS produced by both the host and the pathogens to promote infection [23]. In contrast, plant CAT maintains redox homeostasis by decomposing H_2_O_2_. This process prevents excessive ROS-induced cellular damage, thereby limiting pathogens’ ability to utilize dead host cells for nutrient uptake [24]. Studies have shown that CAT activity is significantly higher in resistant plant genotypes than in susceptible ones [25]. For example, in tomato (*Solanum lycopersicum*), the CAT gene expression is induced by *Alternaria*. Furthermore, *SlCAT* inhibits *Alternaria* infection by regulating H_2_O_2_ homeostasis [26]. The biosynthesis and signaling of JA, a core hormone in defense against necrotrophic pathogens, are regulated by CAT-mediated redox status [27]. In *Arabidopsis*, overexpression of the N-terminal region of CAT2 enhances JA biosynthesis, thereby improving the plant’s resistance to *Botrytis cinerea* strain B05.10. The N-terminal fragment of catalase interacts with fatty acid oxidase, promoting the activity of acyl-CoA oxidase, and thereby enhancing the biosynthesis of JA [28]. These results indicate that *CAT* plays a pivotal role in plant disease resistance.

During the preliminary phase of this experiment, the catalase gene *StCAT1*, which was significantly induced by potato early blight, was identified through transcriptome sequencing. We hypothesized that this gene is involved in the defense response of potato against *A. solani*, although its specific molecular mechanism remains unclear. In this study, we developed an H_2_O_2_ biosensor and found that infection by *A. solani* significantly induces the sustained accumulation of H_2_O_2_ in tobacco leaves. And we confirmed that exogenous application of H_2_O_2_ exacerbates potato early blight infection. Expression analysis of the *StCAT1* gene revealed that its transcription is induced by *A. solani*. To elucidate the biological function of *StCAT1*, we inoculated *StCAT1*-overexpressing and *StCAT1*-silenced plants with *A. solani*. The results demonstrate that *StCAT1* is a positive regulator of potato resistance to early blight. DAB staining was conducted on *StCAT1*-silenced and *StCAT1*-overexpressing leaves. H_2_O_2_ accumulation was increased in silenced leaves and decreased in overexpressing ones. Based on these findings, we further screened and identified StABI5 as an upstream regulator of *StCAT1*. Results from dual-luciferase assays and yeast one-hybrid assays indicate that this transcription factor activates the transcription of *StCAT1*. In summary, StABI5 regulates potato resistance to early blight by activating the expression of *StCAT1* and mediating the ROS signaling pathway, which has laid the theoretical foundation and provided important genetic resources for potato disease-resistant breeding.

## 2. Materials and Methods

### 2.1. Plant Materials and Growing Conditions

The potato variety used in this experiment was ‘Favorita’, with the seed potatoes obtained from the Potato Disease Laboratory of the College of Plant Protection at Hebei Agricultural University. *Nicotiana benthamiana* and common tobacco (*N. tabacum*) were cultivated in greenhouses at Hebei Agricultural University. Relative humidity was maintained at 60% under a 16 h light/8 h dark photoperiod. The cultivar of *N. benthamiana* used was the LAB accession, and the cultivar of *N. tabacum* used was cv. Xanthi nc.

### 2.2. Preparation of Potato Early Blight Spore Suspension and Leaf Inoculation

The HWC168 *A. solani* strain was isolated from potato cultivation areas in China and has undergone whole-genome sequencing [29]. First, the mycelium was inoculated onto a potato dextrose agar (PDA, Product No.: P8931, Beijing, China) medium and cultured at 25 °C for one week. Then, it was transferred to a tomato medium and cultured for another week. After the mycelium covered the entire medium, a sterile scalpel was used to gently scrape off the mycelium in a clean bench. Subsequently, the medium was irradiated with ultraviolet light for 10–20 min in the clean bench. Immediately thereafter, it was cultured alternately in incubators at 20 °C and 25 °C for 3–5 d, with each temperature being maintained for 24 h, to induce sporulation. After the culture was completed, a small amount of sterile water was added to the medium, and the spores were gently scraped off. The spore count was performed using an Olympus BX43 light microscope (Olympus, Tokyo, Japan), employing a 10× eyepiece paired with a 40× objective lens (total magnification of 400×). The spore count was then calculated using a hemocytometer, and the suspension concentration was adjusted to 105 spores/μL for subsequent leaf inoculation experiments, with 20 μL inoculated each time. Select healthy leaves from the silenced, overexpressed, and control groups. Insert leaf petioles into water agar plates. A 20 μL aliquot of spore suspension was applied to a fixed position on the left side of each leaf. Incubate at 25 °C under a 16 h light/8 h dark cycle for 5 d. Measure lesion areas. The potato glucose agar culture medium used in this experiment was purchased from Solarbio (Beijing, China).

### 2.3. Bioinformatics Analysis

The NCBI BLAST tool (https://blast.ncbi.nlm.nih.gov/) was used to identify amino acid sequences highly homologous to the StCAT1 protein sequence of *S. tuberosum*, specifically as SlCAT1 (NP_001234827.1), LbCAT (NP_060173548.1), SvCAT (P55311.1), CaCAT (Q9M5L6.1), LfCAT (XP_059287904.1), SdCAT (XP055811746.1), NtCAT (NP_001312341.1). The structural domains and conservation of these sequences were analyzed in detail by multiple sequence alignment using DNAMAN 8.0.

### 2.4. Probe Construction

Starting from a laboratory-maintained eGFP template, a circularly permuted yellow fluorescent protein (cpYFP) was generated through fragment cloning and fusion PCR. This cpYFP was then fused with various functional components to construct specific H_2_O_2_ probes targeting chloroplasts and mitochondria. H_2_O_2_ biosensors targeting two distinct organelles were transiently expressed in healthy *N. benthamiana* plants via *Agrobacterium*-mediated infiltration. At 48 h of transient expression, the plants were inoculated with *A. solani*. Samples were collected at 0 h, 24 h, 72 h, and 120 h after inoculation with *A. solani*, and fluorescence observation was performed using a Zeiss LSM 710 confocal laser scanning microscopy (Zeiss, Oberkochen, Germany). Excitation wavelengths of 495 nm and 564 nm were used for detecting HyPer-3 and mCherry, respectively.

### 2.5. Potato Plants with stCAT1 Silenced

A 300 bp fragment was amplified using the StCAT1 sequence as a template. Following double restriction, the fragment was inserted into the TRV2 vector and transferred to *Agrobacterium*. *Agrobacterium* containing the StCAT1 fragment, TRV1, and TRV2 was cultured at 28 °C under shaking conditions. With TRV2 serving as the empty vector control, the suspension containing the *StCAT1* construct was mixed 1:1 (*v*/*v*) with the TRV1 suspension and infiltrated into *N. benthamiana* leaves. After one week of culture, viral-containing *N. benthamiana* leaf homogenate was evenly applied to both sides of potato leaves. Following double digestion, the fragment was inserted into the TRV2 vector and introduced into *Agrobacterium*. After four weeks of greenhouse cultivation, *StCAT1* expression levels were measured via RT-qPCR. Plants exhibiting *StCAT1* expression below 50% of the normal level were selected for subsequent disease resistance testing [30].

### 2.6. Tobacco Plants Overexpressing StCAT1

The pCAMBIA2300-*StCAT1* overexpression vector was constructed and *Agrobacterium* was transformed to obtain positive strains. *Agrobacterium* was activated on LB medium containing kanamycin and rifampicin, a bacterial suspension was prepared to an OD_600_ of approximately 0.7, and tobacco leaf discs were infected for 15 min. After rinsing and air-drying, co-cultured in the dark at 28 °C for 2 d. Callus formation, differentiation, and rooting were induced on MS medium to obtain mature plants. Positive plants were screened via RT-PCR. *StCAT1* expression levels were quantified using RT-qPCR combined with the 2^−ΔΔCT^ method, and lines with high expression levels were subsequently selected [31].

### 2.7. Enzyme Activity Assay

This experiment measured the activity of superoxide dismutase (SOD), peroxidase (PAL), and CAT. Following infection of potato leaves with *A. solani*, those with uniform growth stages and comparable sizes were selected for the determination of SOD, PAL, and CAT activity. Enzyme activity was determined using Solarbio enzyme activity assay kits.

### 2.8. DAB Staining

Dissolve DAB to a final concentration of 1 mg/mL in sterile water, adjust the pH to 5.8, and store the solution in the dark. Immerse the entire leaf in the DAB staining solution and incubate at 37 °C in the dark for approximately 15 h. After staining, transfer the leaves to 95% ethanol and heat to decolorize until the leaves become transparent. Inspect the treated leaves for H_2_O_2_ accumulation [32].

### 2.9. Screening of Upstream Regulatory Factors of StCAT1

The promoter sequence of StCAT1 was retrieved from the National Center for Biotechnology Information (NCBI) database. Subsequently, PlantCARE and PlantPAN4.0 were employed to predict its potential upstream regulatory factors.

### 2.10. Yeast Single Hybridization Assay

Ligate StABI5 into pGADT7 and the *StCAT1* promoter into the pHis2 vector. Co-transform the recombinant plasmid containing StABI5 with the recombinant plasmid containing the StCAT1 promoter into yeast strain Y187. Perform the yeast one-hybrid assay according to the instructions of the yeast one-hybrid kit (Coolaber, Beijing, China). Assess yeast growth on selective media SD/Leu/-Trp and SD/-His/-Leu/-Trp [33].

### 2.11. Dual Luciferase Assay

The *StCAT1* promoter was inserted into the PGreenII-0800-LUC vector. *Agrobacterium* suspensions containing the recombinant plasmid pCAMBIA3301-*StABI5* driven by the 35S strong promoter and the recombinant plasmid containing the *StCAT1* promoter were mixed in a 1:1 ratio. The mixture was injected onto the abaxial surface of *N. benthamiana* leaves and incubated at 25 °C for 48–72 h. Acquire images using a fluorescence imaging system after applying fluorescein potassium salt to the leaf abaxial surface. Measure LUC and REN activity using a microplate reader [34].

### 2.12. Statistical Analysis

SPSS 25.0 statistical software was used to process the experimental data, and GraphPad Prism 8.0.1 software was utilized to generate the bar charts shown in this paper. All experimental results were presented as mean ± standard deviation (Mean ± SD). Statistical analysis of differences between the experimental group and control group was performed using independent samples *t*-test at a significance level of *p* < 0.05.

## 3. Results

### 3.1. A. solani Promotes the Accumulation of H_2_O_2_

**Figure 1:**
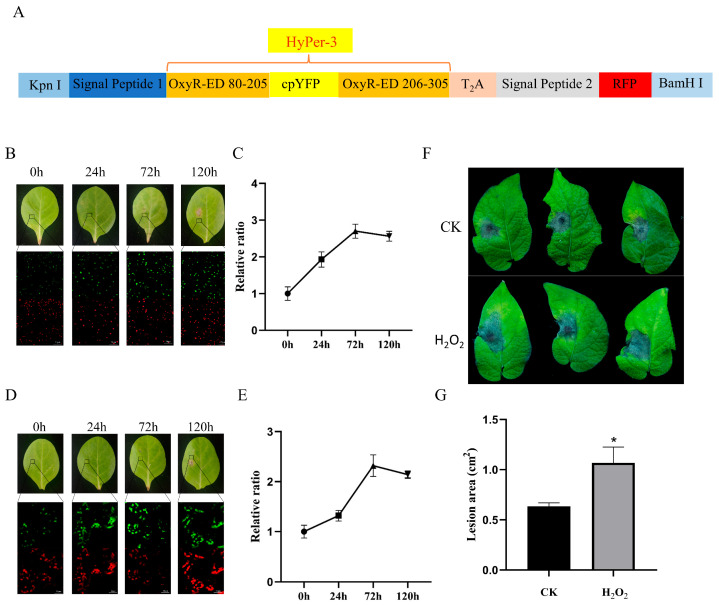
Dynamic changes in H_2_O_2_ levels in chloroplasts and mitochondria after infection by *A. solani* and effects of exogenous H_2_O_2_ on potato infection by the pathogen. (**A**) Schematic diagram of the construction of H_2_O_2_ biosensors targeting chloroplasts and mitochondria. Signal peptide 1 and signal peptide 2 facilitate protein translocation across membranes and targeting to specific organelles, thereby enabling subcellular localization. HyPer-3 is a fluorescent protein probe for detecting H_2_O_2_, used to monitor the dynamic changes of ROS. T_2_A enables the cleavage of the two upstream and downstream proteins, ensuring a 1:1 ratio of the two fluorescent proteins. RFP is a red fluorescent protein used as an internal reference to normalize the fluorescence intensity of HyPer-3, enabling accurate quantification of H_2_O_2_. (**B**,**C**) Relative changes in H_2_O_2_ levels in chloroplasts at different time points (0, 24, 72, 120 hpi) after *A. solani* infection. (**D**,**E**) Relative changes in H_2_O_2_ levels in mitochondria at different time points (0, 24, 72, 120 hpi) after *A. solani* infection. (**F**) Lesion area of potato leaves under control and exogenous H_2_O_2_ treatments after inoculation with *A. solani*. (**G**) Leaf lesions in the H_2_O_2_ treatment group were significantly larger than those in the control group (CK). Statistical significance was assessed using Student’s *t*-test (* *p* < 0.05).

To identify the subcellular sites where *StCAT1* regulates H_2_O_2_, we constructed genetically encoded H_2_O_2_ fluorescent probes that specifically target chloroplasts and mitochondria (Figure 1A). These probes were transiently expressed in *N. benthamiana* leaves to dynamically monitor H_2_O_2_ levels at specific time points during *A. solani* infection (0, 24, 72, 120 hpi) (Figure 1B,D). The results indicate that pathogen infection induces a significant and sustained accumulation of H_2_O_2_ within chloroplasts and mitochondria. In chloroplasts, H_2_O_2_ levels peaked at 72 hpi post-inoculation, representing a 2.70-fold increase relative to the baseline (0 h) (Figure 1C). In mitochondria, H_2_O_2_ accumulation also peaked at 72 hpi, reaching 2.32 times the initial level (Figure 1E). Throughout the entire monitoring period, the accumulation of H_2_O_2_ in chloroplasts consistently exceeded that in mitochondria.

In order to investigate the effect of H_2_O_2_ on the infection of the potato early blight pathogen, potato plants were treated with exogenous H_2_O_2_. The lesion area of H_2_O_2_-treated potato leaves after inoculation was significantly larger than the control. These results show that H_2_O_2_ exacerbates early blight infection in potatoes (Figure 1F,G). These results indicate that *A. solani* infection triggers a significant and sustained accumulation of H_2_O_2_ in tobacco leaves, with the overall accumulation of H_2_O_2_ being higher in chloroplasts than in mitochondria.

### 3.2. Expression Pattern of StCAT1

**Figure 2:**
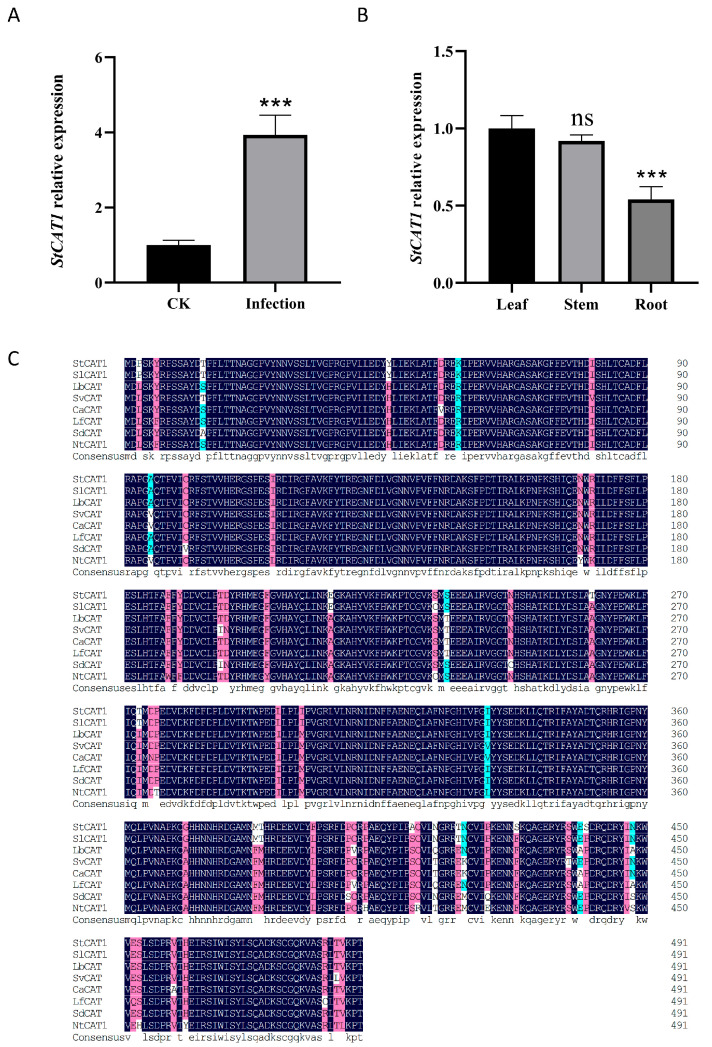
Expression characteristics, tissue specificity and phylogenetic analysis of *StCAT1*. (**A**) Changes in the relative transcript levels of *StCAT1* in potato upon treatment with *A. solani.* The control group consisted of potato plants without *A. solani* inoculation. *StActin7* was used as the reference gene. Significant differences were determined by Student’s *t*-test (*** *p* < 0.001). (**B**) Tissue-specific expression patterns of *StCAT1* in potato (leaves, stems, and roots). There was no significant difference in *StCAT1* expression between leaves and stems (ns, *p* > 0.05), whereas its expression level in roots was significantly lower than that in leaves and stems. Significant difference by Student’s *t*-test, (*** *p* < 0.001). (**C**) A phylogenetic tree was constructed using the homologous *CAT* sequences of *StCAT1* and those from 7 other plant species. The different colors are default display settings of the sequence alignment software. Black indicates identical residues among all eight sequences, pink indicates residues conserved in partial sequences, white indicates residues different from other sequences, and blue indicates non-conserved residues.

To investigate changes in *StCAT1* expression in potato upon infection by *A. solani*, we found that the expression of *StCAT1* was significantly upregulated in potato leaves after inoculation, peaking at 48 hpi. This indicated that the *StCAT1* gene is associated with potato resistance to early blight (Figure 2A). Additionally, the expression of *StCAT1* in different tissues including roots, stems, and leaves was determined by quantitative real-time PCR. No significant difference in *StCAT1* expression levels was found between leaves and stems, whereas the lowest levels were detected in the roots (Figure 2B). The amino acid sequence of *StCAT1* was aligned with homologous sequences from 7 other plant species. The study results demonstrate that StCAT1 exhibits high amino acid sequence similarity with CAT homologs in other Solanaceae species. The catalytic center critical for hydrogen peroxidase activity and the heme-binding motif are fully conserved in StCAT1, indicating that this gene encodes a functional hydrogen peroxidase, and the CAT protein’s ability to scavenge reactive oxygen species is evolutionarily conserved across Solanaceae plants (Figure 2C).

### 3.3. StCAT1 Is a Positive Regulator of Early Blight Resistance in Potatoes

**Figure 3:**
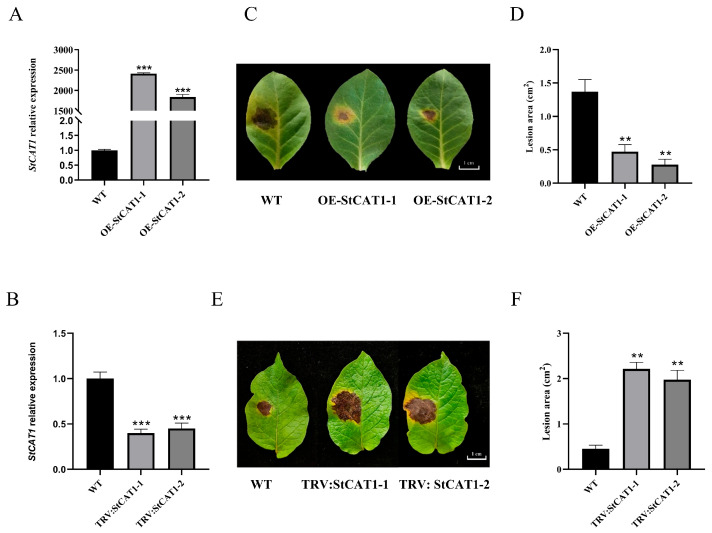
Effects of *StCAT1* overexpression and silencing on plant resistance to *A. solani* infection. (**A**) Relative expression level of *StCAT1* in WT and *StCAT1*-overexpressing tobacco plants. The expression level of *StCAT1* in overexpression lines was significantly higher than that in the wild type. Significant differences were determined by Student’s *t*-test (*** *p* < 0.001). (**B**) Silencing efficiency of *StCAT1* in VIGS-silenced plants. *StCAT1* expression was significantly reduced in the silenced lines. Significant difference by Student’s *t*-test, (*** *p* < 0.001). (**C**) Lesion area in WT and *StCAT1*-overexpressing tobacco plants after inoculation with *A. solani*. (**D**) Quantification of lesion areas on leaves of WT and *StCAT1*-overexpressing tobacco plants at 7 d post-inoculation with *A. solani*. Significant difference by *t*-test (** *p* < 0.01). (**E**) Representative lesion areas on WT and *StCAT1*-overexpressing tobacco leaves following inoculation with *A. solani*. (**F**) Statistical analysis of lesion areas on leaves of control and *StCAT1*-silenced potato plants 5 d after inoculation with *A. solani*. Significant difference by *t*-test (** *p* < 0.01).

To elucidate the biological function of *StCAT1*, we overexpressed *StCAT1* in *N. benthamiana* via *Agrobacterium*-mediated transformation. *StCAT1* expression levels were significantly elevated in *StCAT1*-overexpressing tobacco plants compared to those in WT plants (Figure 3A). No significant differences in growth were observed between the transgenic and WT plants. Additionally, *StCAT1* in potatoes was silenced using virus-mediated gene silencing technology. *StCAT1*-silenced plants were successfully identified by verifying the reduced expression levels of *StCAT1* (Figure 3B). Subsequently, *StCAT1*-overexpressing and *StCAT1*-silenced plants were inoculated with *A. solani* (Figure 3C,E). It was found that 7 d after inoculation, the lesion area in *StCAT1*-overexpressing tobacco plants was significantly smaller than that in WT plants (Figure 3D), whereas at 5 d after inoculation, the leaf lesion area in *StCAT1*-silenced plants was significantly larger than that in control plants (Figure 3F), indicating that Silencing *StCAT1* increases potato susceptibility to early blight. These results indicate that *StCAT1* acts as a positive regulator of resistance to early blight in potato.

### 3.4. StCAT1 Affects H_2_O_2_ Levels and Antioxidant Enzyme Activity

**Figure 4:**
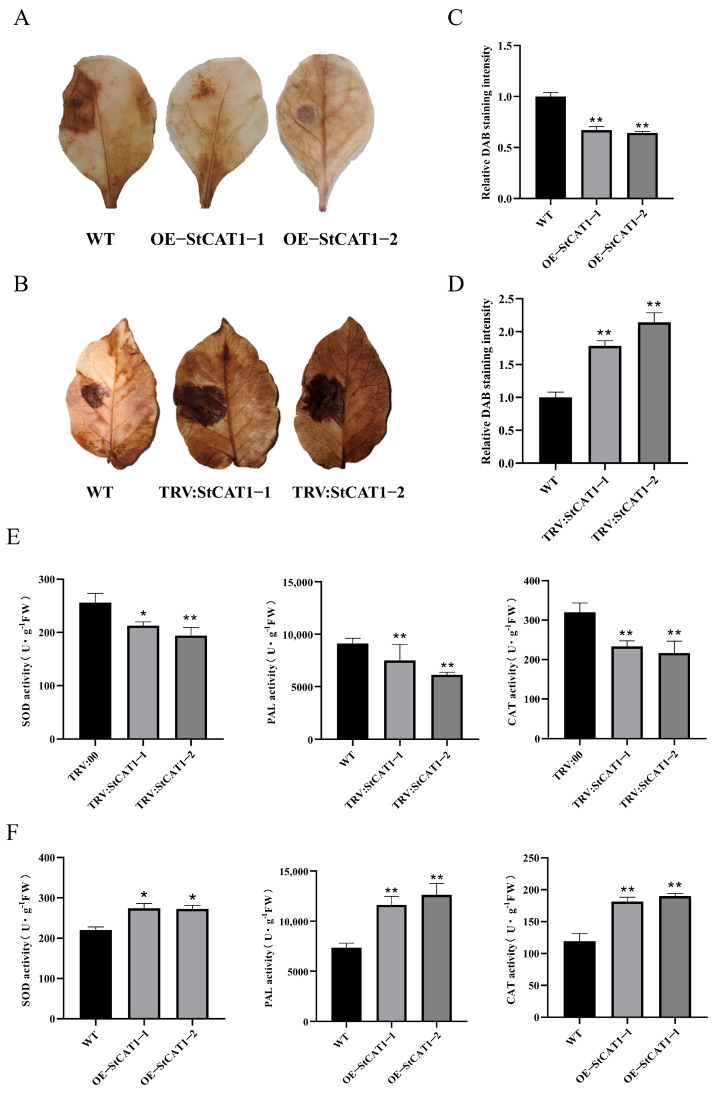
Effects of *StCAT1* overexpression and silencing on H_2_O_2_ accumulation and antioxidant enzyme activities upon *A. solani* infection. (**A**) DAB staining was performed on leaves of WT and *StCAT1*-overexpressing tobacco plants infected with *A. solani*. (**B**) DAB stains hydrogen peroxide (H_2_O_2_), and the brown precipitates (representing H_2_O_2_) in leaves of *StCAT1*-overexpressing plants were significantly less abundant than those in the WT. (**C**) Relative DAB staining intensity in leaves of WT and *StCAT1*-overexpressing tobacco plants. Significant differences by *t*-test (** *p* < 0.01). (**D**) Relative DAB staining intensity in leaves of WT and *StCAT1*-silenced potato plants. Significant differences by *t*-test (** *p* < 0.01). (**E**) Analysis of SOD, PAL and CAT activities in WT and *StCAT1*-silenced potato plants inoculated with *A. solani*. Significant differences by *t*-test (** *p* < 0.01, * *p* < 0.05). (**F**) Analysis of SOD, PAL and CAT activities in WT and *StCAT1*-overexpressing tobacco plants inoculated with *A. solani*. Significant difference by *t*-test (** *p* < 0.01, * *p* < 0.05).

To determine changes in H_2_O_2_ levels following *A*. *solani* infection, DAB staining was performed on plants overexpressing or with silenced *StCAT1* after inoculation with *A. solani* (Figure 4A,B). Tobacco plants overexpressing *StCAT1* exhibited significantly lower H_2_O_2_ levels after fungal inoculation compared to WT plants (Figure 4C), while H_2_O_2_ levels in silenced plants were markedly higher than in WT plants (Figure 4D). To determine whether alterations in antioxidant enzyme activity affect plant resistance to *A. solani*, the activities of SOD, PAL and CAT were analyzed. The results indicate that the activities of SOD, PAL, and CAT in *StCAT1*-overexpressing plants are significantly higher than in WT plants (Figure 4F), while the activities of SOD, PAL, and CAT in silenced plants are significantly lower than in WT plants (Figure 4E).

### 3.5. StABI5 Can Bind to the StCAT1 Promoter and Activate StCAT1 Transcription

**Figure 5:**
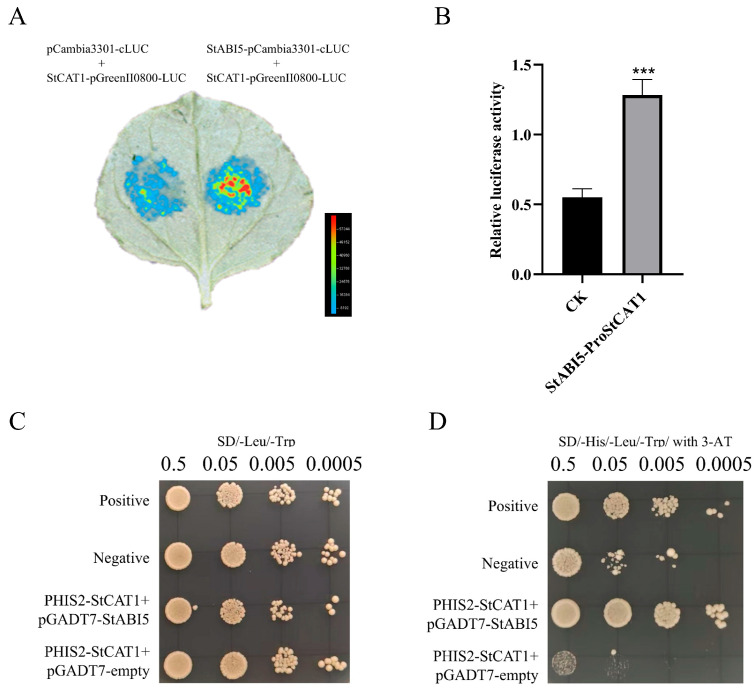
StABI5 binds to and activates the transcription of the *StCAT1* promoter, enhancing plant resistance to early blight. (**A**) The dual luciferase assay shows that StABI5 interacts with the *StCAT1* promoter and activates the transcription of *StCAT1*. 35S:GUS + LUC:*StCAT1* were used as a control, leaf samples were analysed, and luminescence imaging was performed 3 d after infiltration. (**B**) The relative luciferase activity was measured using a microtiter plate reader. Significant differences were determined by Student’s *t*-test (*** *p* < 0.001). (**C**) Yeast one-hybrid assays demonstrated that *StABI5* binds to the promoter of *StCAT1*. (**D**) The growth of pHIS2-*StCAT1* + pGADT7-*StABI5* on SD/−Leu/−Trp/−His medium supplemented with 3-AT was significantly better than that of pHIS2-*StCAT1* + pGADT7-empty.

To further elucidate the regulatory network governing *StCAT1*, we analyzed cis-acting regulatory elements within the potato *StCAT1* promoter sequence. The transcription factor StABI5 was identified as binding to the promoter region. Dual luciferase analysis confirmed that StABI5 could activate the transcription of *StCAT1* (Figure 5A). Results showed that, compared with the control, co-expression of 35S: StABI5 and the LUC reporter driven by the *StCAT1* promoter resulted in significantly higher luminescence intensity and LUC/REN ratios (Figure 5B). To confirm that *StABI5* can bind to the *StCAT1* promoter, we performed a yeast one-hybrid assay. The result indicates that yeast strains co-transformed with pHIS2-*StCAT1* and pGADT7-*StABI5* exhibit similar growth patterns on SD/−Leu/−Trp deficient medium compared to yeast strains co-transformed with pHIS2-*StCAT1* and the empty vector pGADT7-empty (Figure 5C). However, yeast co-transformed with pHIS2-*StCAT1* and pGADT7-*StABI5* exhibited significantly stronger growth on SD/−Leu/−Trp/−His medium containing 3-AT compared to yeast co-transformed with pHIS2-*StCAT1* and pGADT7-empty. This indicates that StABI5 can bind to the promoter of *StCAT1* (Figure 5D). The results indicate that StABI5 can bind to the *StCAT1* promoter and activate its transcription.

### 3.6. StABI5 Plays a Positive Regulatory Role in Potato Resistance to Early Blight

**Figure 6:**
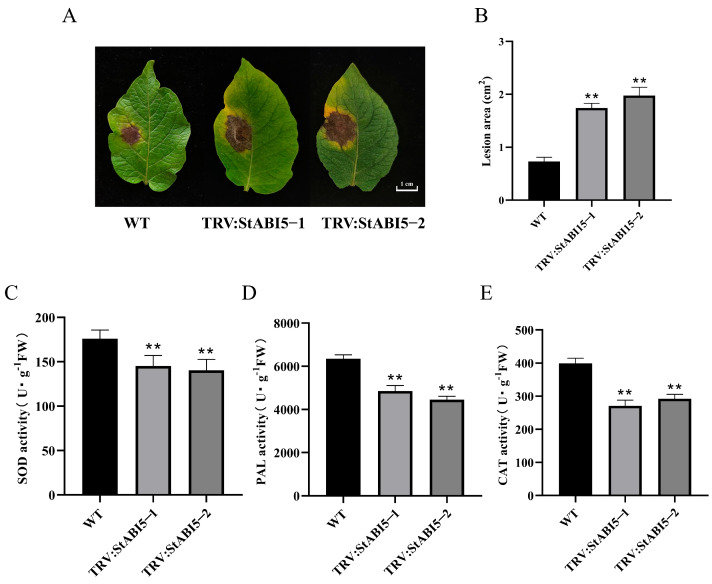
Silencing of *StABI5* in potato increases plant susceptibility to *A. solani.* (**A**) RT-qPCR analysis of the silencing efficiency of the *StABI5* gene in silenced potato plants. (**B**) Statistical analysis of lesion areas on leaves of the control group and *StABI5*-silenced potato plants at 5 d post inoculation with *A. solani.* Significant differences by *t*-test (** *p* < 0.01). (**C**) Analysis of SOD activities in WT and *StABI5*-silenced potato plants inoculated with *A. solani*. Significant differences were determined by Student’s *t*-test (** *p* < 0.01). (**D**) Analysis of PAL activities in WT and *StABI5*-silenced potato plants inoculated with *A. solani*. Significant differences were determined by Student’s *t*-test (** *p* < 0.01). (**E**) Analysis of CAT activities in WT and *StABI5*-silenced potato plants inoculated with *A. solani*. Significant differences were determined by Student’s *t*-test (** *p* < 0.01).

To elucidate the role of *StABI5* in potato resistance to early blight pathogen infection, the *StABI5* silencing vector pTRV2-*StABI5* was constructed using VIGS technology. The silencing efficiency of *StABI5* in potato plants was assessed by RT-qPCR. Leaves of *StABI5*-silenced and control potato plants were inoculated with *A. solani* (Figure 6A). Five days after inoculation, the leaf lesion area in *StABI5*-silenced plants was significantly larger than that in control plants (Figure 6B). This result indicates that silencing *StABI5* reduces potato resistance to early blight. Subsequently, the activities of antioxidant enzymes (SOD, PAL, CAT) were analyzed. The results showed that the activities of these antioxidant enzymes were significantly lower in *StABI5*-silenced plants compared to the controls (Figure 6C–E). These results suggest that *StABI5* acts as a positive regulator in potato resistance to early blight.

## 4. Discussion

CAT, as one of the core antioxidant enzymes maintaining redox homeostasis in plants, is widely distributed across all aerobic organisms. Its gene family typically exhibits a multi-copy characteristic in plant genomes. Numerous studies indicate that altered expression levels of CAT serve as a key indicator of plant responses to external stress. Plants counteract infections by various pathogens, including bacteria, fungi, and viruses, through the efficient expression of *CAT* genes [35,36].

In plant stress biology, the expression of host plant *CAT* genes is regulated by environmental stress. Analysis of transcriptome data from previous laboratory studies on *A. solani* infection in potatoes revealed that *StCAT1* expression was significantly upregulated following pathogen challenge. Sequence alignment confirmed that potato *StCAT1* shares high sequence homology with tomato *SlCAT1*. Research by Kabir and Wang demonstrates that tomato *SlCAT1* maintains ROS homeostasis by specifically scavenging H_2_O_2_, thereby enhancing plant resistance to environmental stress [37]. Similarly, Zhang et al. reported that overexpression of *GmCAT1* enhances soybean resistance to *Phytophthora soyae* [38]. In this study, *A. solani* was employed to inoculate potato plants with silenced *StCAT1* and tobacco plants overexpressing *StCAT1*. Results showed that *StCAT1*-silenced plants exhibited increased susceptibility, while *StCAT1*-overexpressing plants demonstrated significantly enhanced resistance. These findings suggest that *StCAT1* positively regulates plant resistance against *A. solani*.

H_2_O_2_ acts as a pivotal molecule in plant defense against environmental stress. It not only functions as an early signaling molecule to activate downstream defense signaling pathways but also leverages its potent oxidative capacity to induce toxic effects that block pathogen invasion [39]. However, the role of H_2_O_2_ in plant defense against saprophytic pathogens is more complex. Although it activates disease resistance pathways, such as the JA and ET pathways, its strong oxidative potential can simultaneously induce host cell death, consequently facilitating infection by necrotrophic pathogens. Therefore, maintaining redox homeostasis within host cells is crucial for resisting pathogen invasion [40].

Research indicates that CAT significantly reduces intracellular toxic ROS concentrations by specifically catalyzing the decomposition of H_2_O_2_ into H_2_O and O_2_. This process inhibits the oxidation of membrane lipids and damage to biomolecules, thereby safeguarding the integrity of cellular structure and function [41]. Kuzniak and Sklodowska reported that gray mold pathogens promote increased H_2_O_2_ content by suppressing the expression of the *CAT* gene in tomatoes, thereby facilitating pathogen infection [42]. Furthermore, reports have demonstrated that the black spot pathogen can promote infection by elevating endogenous ROS levels in barley [43]. Using DAB staining, this study detected H_2_O_2_ accumulation in the leaves of *S. tuberosum* inoculated with *A. solani*. Results indicate that susceptible *StCAT1*-silenced plants showed higher H_2_O_2_ levels than controls, whereas resistant *StCAT1*-overexpressing plants exhibited lower H_2_O_2_ levels than WT plants. Furthermore, the level of antioxidant enzyme activity is crucial for plant resistance against saprophytic pathogens [44]. For example, after infection of tomato leaves by *B*. *cinerea*, the activity of the peroxisomal antioxidant system shows a staged increase, indicating that plants counteract pathogen-related oxidative stress by synergistically activating a multi-enzyme system [45]. In contrast, *Fusarium* spp. can also improve their infection success by inhibiting the host’s antioxidant enzyme activity when infecting barley [46].

Previous studies indicate that increased CAT, SOD, and PAL enzyme activities can enhance plant disease resistance [47]. This study examined changes in plant resistance to *A. solani*. Transgenic tobacco plants overexpressing *StCAT1* exhibited elevated CAT, SOD, and PAL enzyme activities, demonstrating enhanced resistance to the pathogen. In contrast, following functional suppression of the *StCAT1* gene, CAT, SODand PAL enzyme activities decreased in potatoes, resulting in increased susceptibility to the pathogen.

This study isolated the *StCAT1* promoter sequence via PCR amplification. Following successful cloning and sequencing, the promoter was analyzed using PlantCARE and PlantPAN4.0, revealing that the *StCAT1* promoter contains numerous ABRE cis-acting elements that bind to bZIP proteins [48]. Analysis of existing laboratory transcriptome data from potato plants infected with the early blight pathogen identified transcription factors exhibiting expression patterns similar to those of the *StCAT1* gene. Subsequent functional analysis of transcription factors identified six candidate transcription factors as potential regulators of *StCAT1* expression: StERF-1B, StNAC1, StbZIP9, StAThook-28, StAHL-17, and StABI5. The dual-luciferase reporter assay further confirmed that StABI5 positively activates the *StCAT1* promoter and enhances LUC expression. These results suggest that StABI5 functions as a positive transcriptional regulator of *StCAT1*. StABI5 belongs to the bZIP transcription factor family and serves as a core transcription factor in ABA signaling, regulating canonical ABA-mediated processes such as seed germination and stress responses [49]. Current reports on ABI5 indicate that it does not function as a typical disease resistance gene. Research by Bi et al. demonstrates that *Arabidopsis* ABI5 can bind to the *CAT1* promoter and modulate ROS homeostasis, thereby regulating seed germination and resistance to external stressors [50]. However, related literature also reports that ABI5 can mediate the balance between SA and JA by regulating ABA, thereby influencing plant resistance to pathogens [51]. Recent reports on gibberellic acid regulatory pathways indicate that ABI5, which interacts with ABA-regulated DELLA proteins, participates in the regulatory pathways of JA and SA [52]. Furthermore, our laboratory’s prior research demonstrates that SA can engage in crosstalk with other plant hormones (synergistic responses with ABA and brassinosteroids, and initially antagonistic followed by synergistic responses with JA), thereby enhancing plant resistance to *A. solani*. Following StABI5 silencing in this experiment, potato resistance to *A. solani* was significantly reduced. Since *CAT* also mediates complex regulatory pathways involving JA, SA, and H_2_O_2_, and given the limited *StABI5*-related validation experiments in this study, further experimental verification of its regulatory mechanism is required.

## 5. Conclusions

This study elucidates the molecular mechanism of potato response to *A*. *solani* infection. The results show that *A*. *solani* infection induces hydrogen peroxide accumulation in plants, and exogenous H_2_O_2_ application markedly accelerates early blight infection. Gene expression and functional validation demonstrated that *A. solani* induces *StCAT1*, which positively regulates antioxidant enzymes (SOD, PAL, CAT) and confers positive regulation of early blight resistance through ROS signaling. Yeast one-hybrid and dual-luciferase assays confirmed that the transcription factor StABI5 can target and activate the expression of *StCAT1*, thereby enhancing disease resistance in potato. In conclusion, the StABI5–*StCAT1* module mediates ROS signaling to modulate potato resistance against early blight in this study, providing novel genetic resources and theoretical basis for molecular breeding of potato resistance to early blight.

## Data Availability

All data supporting the findings of this study are available within the article. Further inquiries should be addressed to the corresponding authors.

## Abbreviations

The following abbreviations are used in this manuscript:

ROS	Reactive oxygen species
JA	Jasmonic Acid
SA	Salicylic Acid
ABA	Abscisic acid
SOD	Superoxide Dismutase
PAL	Peroxidase
CAT	Catalase
